# Translating Marine Symbioses toward Drug Development

**DOI:** 10.1128/mbio.02499-22

**Published:** 2022-10-31

**Authors:** Eric W. Schmidt, Zhenjian Lin

**Affiliations:** a Department of Medicinal Chemistry, University of Utah, Salt Lake City, Utah, USA

**Keywords:** drug development, marine microbiology, metagenomics, natural products, symbiosis

## Abstract

Chemists have studied marine animals for the better part of a century because they contain a diverse array of bioactive compounds. Tens of thousands of compounds have been reported, many with elaborate structural motifs and biological mechanisms of action found nowhere else. The challenge holding back the field has long been that of supply. Compounds are sometimes obtained by cultivating marine animals or by wild harvest, but this often presents logistical and environmental challenges. Some of the most medically important marine animal compounds are supplied by synthesis, often through multistep procedures that delay drug development. A relatively small number of such agents have been approved by the U.S. Food and Drug Administration, often after a heroic effort. In a recent *mBio* paper, Uppal and coworkers (https://doi.org/10.1128/mBio.01524-22) address key hurdles underlying the supply issue, discovering an uncultivated new bacterial genus from a marine sponge and reconstituting the biosynthetic pathway for expression.

## COMMENTARY

The paper by Uppal et al. in *mBio* (1) focuses on lasonolide, a compound from the macrocyclic polyketide class of natural products. Such polyketides are an especially important group of compounds and illustrate the progression of the field. Early promising marine animal drug leads included bryostatins (from a bryozoan) (2) and halichondrin (from a sponge) (3). Both compounds were discovered by grinding up and extracting the animals with solvents, followed by extensive spectroscopic and chemical studies. An immense effort was required to supply the compounds. Aquaculture was attempted for both compounds with some success, but ultimately, chemical synthesis provided the supply used for drug development. Halichondrin was simplified in a medicinal chemistry effort, leading to the approved cancer drug eribulin. While not yet approved, the synthesis of bryostatin has led to numerous clinical trials for cancers and other diseases. These heroic efforts are difficult to replicate for every promising marine compound.

Bryostatin also holds a key place in marine symbiosis. Like other potent compounds found in benthic marine invertebrates, bryostatin’s toxicity affords it with a striking property: it defends the animal from predation (4). It had long been somewhat controversially hypothesized that marine animals do not usually produce defensive compounds themselves but, instead, that symbiotic bacteria are often the true producers. In the early 1990s, experimental evidence supporting this idea was gathered for several systems, but it was bryostatin where the first convincing molecular evidence was obtained. In 1997, Seana Davidson and Margo Haygood discovered an uncultivated bacterium that lived in close symbiosis with the host animal (5). The team obtained the first bacterial polyketide synthase (PKS) genes from an uncultivated symbiotic consortium in work that provided direct evidence that symbiotic bacteria produce defensive compounds.

The implications of this work were staggering. The tight symbiosis between animals and bacteria may be built upon the chemical defense of the holobiont, leading to a new field of inquiry. The work also prophesied a new era in marine drug development. Perhaps, instead of total synthesis, we might eventually learn to cultivate the uncultivable. Alternatively, although well beyond current art at the time, perhaps taking a biotechnological approach, moving the biosynthetic genes to a cultivated host, might solve the supply problem.

This early promise has required decades of work and is still only approaching fruition. Several PKS pathways to important bioactive compounds have since been identified in marine animals and also in other symbiotic systems, such as insects, and in the human microbiome. The most significant early step was reported by Jörn Piel, who focused on the production of the highly toxic defensive metabolite, pederin, from a beetle (6). In 2002, Piel created a large-insert library from the beetle and its bacterial symbiont. He sequenced a large portion of a PKS-nonribosomal peptide synthetase (NRPS) gene cluster from the uncultivated symbiont and convincingly associated it with production in the uncultivated bacterium by using a sequence- and bioinformatics-based argument. Piel and coworkers then reported a homologous pathway in uncultivated bacterial symbionts from a marine sponge. Analysis of these chemical symbioses ultimately moved away from making plasmid libraries to metagenome sequencing and assembly.

In their recent mBio paper (1), a collaborative effort between Jason Kwan and Guojun Wang labs led to the discovery of a symbiotic bacterium that produces lasonolides in sponges. Lasonolide A was discovered in Oliver McConnell’s group in 1994 from a Caribbean sponge, *Forcepia* sp. (7). Its potent subnanomolar toxicity has led to decades of ongoing studies aiming toward drug development. Total syntheses have led to a supply of the compound, but their complexity still presents challenges in terms of development. More recent work has demonstrated a very unusual biological mechanism underlying lasonolide anticancer action, reinvigorating efforts at drug development (8). This new paper describes an innovative biotechnological approach to obtaining a large biosynthetic gene cluster (BGC) required for producing lasonolide, setting the stage for heterologous production in a cultivated bacterial strain.

The Kwan and Wang groups used a *Forcepia* sp. sponge originally obtained by submarine in the Caribbean. They employed a hybrid strategy of obtaining both a genomic DNA fosmid library and performing direct metagenome sequencing from two different tissues. This proved crucial in the accurate capture of the full lasonolide BGC. Based on the chemical structure of lasonolide, the authors hypothesized that a *trans*-acyltransferase (AT) pathway was involved in biosynthesis. This enabled the design of a PCR-based method to identify fosmids encoding a part of the BGC. Unfortunately, only about half of the required genes were identified in the library. A BLAST search of the assembled metagenome led to the discovery of most of the lasonolide BGC (*las*), the sequence of which could then be used to fish out the remainder of the pathway from the fosmid library. In turn, sequencing of the fosmids was crucial to the accurate assembly of *las.* The *las* BGC had several challenging features (detailed below) that made it an especially difficult analytical problem, which was resolved with the help of the accurate long sequences available from the fosmids. The acquisition of fosmids was also critically important because the fosmids were assembled into a single intact BGC for expression in a cultivable host organism, especially impressive given the large size of the BGC (>100 kbp). Overall, this series of steps shows promise in solving critical bottlenecks en route to marine animal drug development.

The *las* BGC presented several difficult challenges in bioinformatics analysis. These were circumvented in a series of creative methods developed in the work. One of the crucial steps in metagenome analysis is binning, in which pieces of assembled DNA (contigs) are sorted into bins representing different biological origins, such as different bacterial species. However, contigs containing *las* had a much different GC bias and k-mer coverages than the producing bacterial strain as a whole, leading some contigs to be sorted into the wrong bin. This was proposed to result from a recent horizontal gene transfer (HGT). To overcome this challenge, the authors used assembly graph examination and paired-end (PE) reads mapping to correct binning mistakes. Automappa, developed by the Kwan group, also drastically simplified the binning correction process.

Another informatics challenge resulted from repetitive sequences in the *las* BGC. Tools such as metaSPAdes are widely used in metagenome assembly, but repetitive sequences compromise their ability to assemble and scaffold sequencing data. The *las* BGC was present in three nearly identical copies in a single genome, complicating assembly efforts. Some regions had a much deeper coverage than others. These three repeats were assembled using assembly graph and PE reads mapping. PCR experiments confirmed the linkages between these putative assembled BGCs. No mutations in the three BGCs were detected that might affect the function of the genes, and thus, the authors proposed that *las* is one of the few known examples in which repeated BGCs might increase the titer of a natural product in bacteria.

The major defensive chemicals in marine animals are often encoded in the genomes of symbiotic bacteria that are only distantly related to known or cultivated strains. Here, the authors found that the producing bacterium, “*Candidatus* Thermopylae lasonolidus,” was only 88.78% identical in its 16S rRNA gene sequence to its closest relative, making it novel at the genus level. It is one of the growing number of *Verrucomicrobia* known to be involved in defensive symbioses in animals. The availability of the high-quality binning analysis enabled the assembly of a nearly complete genome and the analysis of key features of the symbiont. Among these, it was notable that about 16% of genes in the bacteria were pseudogenes, potentially indicating the early stages of genome reduction that are sometimes observed in bacterial symbioses.

This work has a few limitations. The identification of the *las* pathway was performed using strong bioinformatics analysis in which every biosynthetic step could be accounted for in the identified genes, but no functional analysis was yet performed. This remains a limiting challenge in connecting metagenome sequencing to chemistry. While the large *las* BGC was completely assembled in a plasmid for expression, those expression experiments have not yet been described. As of this writing, however, there are no functional heterologous expression experiments of large PKS pathways from these types of symbiosis that we are aware of. Thus, significant challenges remain to fully develop these promising new methods.

Overall, this work represents an excellent example of obtaining and classifying a high-quality metagenome-assembled genome (MAG) from a highly complex sample. The methods presented here will be highly useful in the discovery of further biosynthetic pathways from symbiotic associations, in particular those in which the potential biosynthetic genes or enzymes are difficult to predict. This should greatly streamline the early steps in biotechnological approaches to the supply problem, which, in the future, will speed the development of promising marine natural products. Moreover, there are still many marine and other animals with unknown chemistry that may be promising as pharmaceuticals to serve human health needs. Many animals are limited in size and rare or difficult to find, so chemical studies are challenging. The metagenome mining strategy described in this work opens new possibilities to uncover their pharmaceutical potential.
